# Speaking in Public Coping Scale (ECOFAP): validity evidence based on internal structure

**DOI:** 10.1590/2317-1782/e20240309en

**Published:** 2025-12-08

**Authors:** Anna Carolina Ferreira Marinho, Adriane Mesquita de Medeiros, Eduardo de Paula Lima, Letícia Caldas Teixeira

**Affiliations:** 1 Programa de Pós-graduação em Ciências Fonoaudiológicas, Universidade Federal de Minas Gerais – UFMG - Belo Horizonte (MG), Brasil.; 2 Departamento de Fonoaudiologia, Faculdade de Medicina, Universidade Federal de Minas Gerais – UFMG - Belo Horizonte (MG), Brasil.; 3 Corpo de Bombeiros Militar de Minas Gerais - Belo Horizonte (MG), Brasil.

**Keywords:** Validation Study, Coping, Public Speaking, Self-assessment, Psychometrics

## Abstract

**Purpose:**

To present the validity evidence of the Speaking in Public Coping Scale (ECOFAP) based on its internal structure.

**Methods:**

This methodological study of instrument development and validation included 1,119 adults who speak in public in academic or professional contexts, with a mean age of 25 years. Most of the sample were females (68.5%) and held a bachelor’s degree (58.5%). A self-report questionnaire was used for validation, including: 1) sociodemographic characteristics (age, sex, education, profession); 2) questions about participation in training to improve public communication and public speaking experience in academic and professional contexts; 3) the 48-item Speaking in Public Coping Scale (ECOFAP). Internal consistency analyses (Cronbach's alpha), exploratory factor analysis, and confirmatory factor analysis were performed for the validity evidence stages.

**Results:**

ECOFAP had 30 items after finishing the analyses. The results indicated it had good internal consistency (Cronbach's alpha 0.87). The instrument presented a factorial structure comprising the Challenge (13 items) and Threat (17 items) factors. Factor loadings ranged from 0.37 to 0.66.

**Conclusion:**

ECOFAP has a solid internal structure and adequate psychometric properties. It is reliable and valid for self-assessing coping strategies for public speaking among adults in academic and professional contexts.

## INTRODUCTION

The Speaking in Public Coping Scale (ECOFAP) is a self-assessment instrument designed to assess adults' self-regulation strategies during public speaking. Its development followed the criteria established by the manual of the Standards for Educational and Psychological Testing (SEPT), which defines the methodological steps for developing and validating measurement instruments. They include obtaining validity evidence based on the test's content, response processes, internal structure, relationship with other variables, and test outcomes^(1)^.

ECOFAP was developed based on the theoretical frameworks of public speaking^(2)^ and the Motivational Coping Theory (MCT)^(3-5)^. Public speaking is understood as the ability to orally convey a message to an audience in various contexts, whether academic, social, or professional^(6,7)^. In this communicative activity, the individual faces challenges such as developing a coherent speech, informing and entertaining the audience, adapting the message to the target audience, presenting oneself clearly and confidently, and dealing with nervousness and fear of exposure^(7-11)^.

MCT is a developmental approach to stress coping, based on the approach of coping as a regulatory action of one's own behavioral patterns, motivations, and emotions in the face of stressful conditions^(3-5)^. The stressful event is perceived as a challenge or threat to the basic psychological needs of relatedness, competence, and autonomy^(3-5,12,13)^. The theory describes 12 coping strategies/families, according to a positive adaptive outcome (challenge): self-confidence, seeking support, solving problems, seeking information, adapting, and negotiating; or a negative outcome (threat): delegation, isolation, helplessness, avoidance, submission, and opposition^(3-5)^.

It is believed that personal awareness of coping strategies for public speaking can help people better manage communication situations. This knowledge can help to develop specific interventions and counseling to improve communication and promote emotional and mental well-being during public speaking activities.

ECOFAP was validated in two stages according to instrument validation guidelines^(1)^. The first, called content and response process validation, already published in a previous article, demonstrated that the items have adequate semantic and syntactic structures to represent the constructs of public speaking and coping^(14)^. Following the recommendations of SEPT^(1)^, this article presents the second stage: internal structure validation, which assesses the internal consistency, validity, and reliability of the measures. Hence, this study aimed to present evidence of the validity of the ECOFAP based on its internal structure.

## METHODS

### Study type

Methodological study of development and validation of a measuring instrument, approved by the Research Ethics Committee, under approval number 5,735,670/2022.

### Participants

Adults of both sexes were invited to participate in the study. The following inclusion criteria were established: having experienced public speaking situations in academic and occupational contexts and being at least 18 years old. Psychology and speech-language-hearing students and professionals and those who had previously participated in any training in public speaking were excluded from the sample to avoid selection bias. Participants were selected through non-probability sampling, using a snowball approach. Initially, 1,190 individuals responded to the survey, of whom 71 were excluded due to the established eligibility criteria. The final sample consisted of 1,119 adults, including 308 university students, 250 lawyers, 253 entrepreneurs, 100 university professors, 100 executives, 61 speakers, and 46 politicians.

### Recruitment of participants

The survey was disseminated in various online environments, including speech-language-hearing therapy groups specializing in communication improvement, Instagram profiles, institutional emails from a Brazilian public university, human resources departments of technology companies, law firms, and political offices. Data were collected over 4 months using Google Forms. Participants received an invitation letter containing a link to an informed consent form and a questionnaire with the assessment tool.

### Validation instrument

The questionnaire inquired into sociodemographic characteristics (age, sex, education, profession), a question about participation in communication training, and a question about public speaking experience in professional or academic contexts. The questionnaire also included the ECOFAP. The response key was a Likert-type scale ranging from 1 (strongly disagree) to 5 (strongly agree). The pilot ECOFAP had 48 items, including the 12 coping categories/families: solving problems, seeking information, helplessness, avoidance, self-confidence, seeking support, delegation, isolation, adapting, negotiating, submission, and opposition. The items were randomly assigned to the participants and did not indicate the families they represented.

### Data analysis

A descriptive analysis of sociodemographic variables was performed. Then, analyses of the psychometric properties of ECOFAP, reliability measures, and dimensionality were conducted using the Cronbach's alpha coefficient, exploratory factor analysis (EFA), and confirmatory factor analysis (CFA).

Cronbach's alpha was calculated to assess the internal consistency of the ECOFAP, considering values ​​> 0.60 as indicative of good reliability^(15-18)^. The viability of the EFA was verified by the Kaiser-Meyer-Olkin (KMO) index, considering adequate values ​​> 0.70, and by Bartlett's Test of Sphericity, with p-values < 0.05. The EFA followed principal components analysis, adopting the Kaiser criterion, which maintains factors with eigenvalues ​​> 1. Factor loadings were extracted using the Varimax orthogonal rotation method, considering adequate values > 0.30. The analysis was performed using the Statistical Package for the Social Sciences (SPSS), version 26.

The EFA was conducted in two stages. In the initial stage, 48 items were evaluated, four in each coping family. To reduce the measurement instrument and following the theoretical assumption of maintaining three items per coping family, the authors removed one item from each ECOFAP domain. The criteria used for removal were items with similar semantic structure and factor loadings within the same domain, and items with factor loadings below the values ​​recommended in the literature. Based on the EFA results, the CFA was then performed using the structural equation modeling (SEM) program.

The following criteria were considered to adjust the model: normed chi-square x^2^/df (values ​​between 1 and 5); normality of fit index GFI (> 0.90); adjusted goodness of fit index AGFI (> 0.90); root mean square error of approximation RMSEA (0.05-0.10); Tukey-Lewis index TLI (> 0.90)19; comparative fit index CFI (> 0.90)^(18,19)^. This analysis was performed using Stata Corp, College Station, STATA, version 12.

## RESULTS

The participants' ages ranged from 18 to 66 years, with a mean of 22 years. The majority were female (n = 697, 68.5%) and had a bachelor’s degree (n = 596, 58.5%). The principal components analysis (eigenvalues) and the variance of the 12 ECOFAP factors are shown in Table 1. The eigenvalues ​​ranged from 1.60 (solving problems) to 3.01 (avoidance), and the variance ranged from 53.42 (solving problems) to 89.49 (submission).

**Table 1:** Analysis of the main ECOFAP components

ECOFAP factors	Self-values	% variance
Solving problems	1.60	53.42
Seeking information	1.95	65.1211
Self-confidence	1.98	66.11
Seeking support	2.24	55.96
Adapting	1.70	56.74
Negotiating	1.80	60.25
Helplessness	2.43	81.17
Avoidance	2.39	79.76
Delegation	3.01	75.33
Isolation	2.61	65.29
Submission	2.47	89.49
Opposition	2.12	70.79

The internal structure of the ECOFAP, proposed in the EFA, is shown in Table 2. The process was carried out in two stages. The first stage used the 48-item pilot version of the ECOFAP for initial analysis. Item-total correlation coefficients ranged from 0.08 to 0.76. Cronbach's alpha values ​​ranged from 0.57 to 0.90, and factor loadings ranged from 0.14 to 0.66. Twelve items were removed based on their values ​​of the item-total correlation coefficients, Cronbach's alpha, and factor loadings, following the theoretical assumption of the CMT of maintaining three items per coping family. From this analysis, seven items had low item-total correlation coefficients (items 3, 8, 14, 20, 21, 25, 40), one (item 21) had a non-significant factor loading, and five (items 11, 32, 36, 41, 48) were excluded as they increased the Cronbach's alpha values ​​of their respective domains and had lower factor loadings within the same domain. After excluding these 12 items, a new ECOFAP was analyzed with 36 items. In this second stage, all parameters were statistically significant. The item-total correlation coefficients ranged from 0.30 to 0.090, Cronbach's alpha ranged from 0.60 to 0.89, and the factor loadings had weights ranging from 0.37 to 0.66.

**Table 2:** ECOFAP exploratory factor analysis – stage 1: 48-item ECOFAP; stage 2: 36-item ECOFAP

Domains and Items	STAGE 1: 48-items ECOFAP			STAGE 2: 36-items ECOFAP		
	Item-total correlation	*a* if item is excluded	Factor loading	Item-total correlation	*a* if item is excluded	Factor loading
**Solving problems**		0.59			0.60	
**1**	0.50	0.45	0.57	0.51	0.60	0.63
**2**	0.31	0.51	0.53	0.30	0.44	0.61
3 ***	0.20	0.60	0.48	-	-	-
**4**	0.50	0.58	0.39	0.49	0.55	0.46
**Seeking information**		0.73			0.72	
**5**	0.30	0.65	0.52	0.31	0.53	0.62
**6**	0.30	0.69	0.48	0.32	0.82	0.45
**7**	0.30	0.62	0.54	0.30	0.65	0.63
8 ***	0.21	0.72	0.44	-	-	-
**Self-confidence**		0.75			0.76	
**9**	0.70	0.72	0.46	0.90	0.75	0.52
**10**	0.34	0.65	0.54	0.33	0.66	0.61
11***	0.37	0.76	0.46	-	-	-
**12**	0.70	0.68	0.52	0.70	0.68	0.52
**Seeking support**		0.72			0.60	
**13**	0.31	0.75	0.36	0.31	0.60	0.61
14 ***	0.26	0.60	0.57	-	-	-
**15**	0.32	0.72	0.44	0.32	0.43	0.48
**16**	0.32	0.56	0.58	0.31	0.31	0.59
**Adapting**		0.59			0.60	
**17**	0.34	0.43	0.58	0.34	0.39	0.63
**18**	0.44	0.45	0.57	0.43	0.44	0.62
**19**	0.30	0.57	0.42	0.30	0.65	0.61
20 ***	0.08	0.60	0.38	-	-	-
**Negotiating**		0.57			0.65	
21 ***	0.17	0.65	0.14	-	-	-
**22**	0.33	0.50	0.37	0.31	0.80	0.47
**23**	0.64	0.36	0.66	0.64	0.34	0.37
**24**	0.72	0.39	0.65	0.72	0.39	0.66
**Helplessness**		0.90			0.88	
25 ***	0.22	0.88	0.49	-	-	-
**26**	0.72	0.87	0.50	0.73	0.87	0.55
**27**	0.65	0.87	0.50	0.64	0.80	0.58
**28**	0.69	0.87	0.50	0.69	0.80	0.58
**Avoidance**		0.90			0.84	
**29**	0.73	0.85	0.51	0.73	0.72	0.60
**30**	0.73	0.85	0.52	0.73	0.71	0.60
**31**	0.62	0.92	0.42	0.62	0.89	0.52
32 ***	0.76	0.84	0.52	-	-	-
**Delegation**		0.88			0.86	
**33**	0.67	0.86	0.49	0.66	0.80	0.58
**34**	0.71	0.84	0.50	0.71	0.87	0.55
**35**	0.74	0.84	0.51	0.74	0.76	0.59
36 ***	0.69	0.86	0.48	-	-	-
**Isolation**		0.82			0.79	
**37**	0.46	0.77	0.50	0.46	0.66	0.60
**38**	0.48	0.76	0.51	0.48	0.62	0.61
**39**	0.67	0.77	0.49	0.67	0.85	0.50
40 ***	0.25	0.79	0.47	-	-	-
**Submission**		0.89			0.89	
41***	0.59	0.89	0.47	-	-	-
**42**	0.67	0.84	0.52	0.65	0.82	0.58
**43**	0.69	0.87	0.50	0.69	0.85	0.57
**44**	0.60	0.86	0.49	0.61	0.85	0.57
**Opposition**		0.77			0.79	
**45**	0.32	0.71	0.50	0.33	0.75	0.56
**46**	0.32	0.68	0.53	0.34	0.67	0.59
**47**	0.40	0.68	0.53	0.41	0.71	0.57
48 ***	0.46	0.79	0.40	-	-	-

*** item excluded from the instrument; *a* = Cronbach’s alpha coefficient

Table 3 presents the SEM adjustment indices. The initial GFI, AGFI, RMSEA, TLI, CFI, and PGFI values are below the minimum recommended in the literature19, failing to confirm the ECOFAP factorial structure with 12 factors. The authors decided to test a new two-factor structure, grouping six families of coping with a focus on challenge (solving problems, seeking information, self-confidence, seeking support, adapting, and negotiating) and six with a focus on threat (helplessness, avoidance, delegation, isolation, submission, and opposition). As previously pointed out, the values ​​were adequate. In this process, it was necessary to exclude items 24 (0.11), 23 (0.13), 1 (0.15), 19 (0.19), 45 (0.28), and 2 (0.29), starting with the lowest value because the factor loadings were below the 0.30 significance level.

**Table 3:** Structural Equation Modeling adjustment indicators for ECOFAP validation

Adjustment indicators	Final model
**12-factor ECOFAP**	
Discrepancy function: x^2^ (p-value)	29.380
Normed chi-square (x^2^/df)	0.59
GFI (goodness-of-fit index)	0.78
AGFI (adjusted goodness-of-fit index)	0.73
RMSEA (root mean square error of approximation)	0.50
TLI (Tukey-Lewis Index)	0.85
CFI (comparative fit index)	0.78
**2-factor ECOFAP**	
Discrepancy function: x^2^ (p-value)	4392.291
Normed chi-square (x^2^/df)	1.73
GFI (goodness-of-fit index)	0.96
AGFI (adjusted goodness-of-fit index)	0.93
RMSEA (root mean square error of approximation)	0.05
TLI (Tukey-Lewis Index)	0.96
CFI (comparative fit index)	0.92

The multifactorial structure considering two factors for ECOFAP is presented in Table 4. It was found that the challenge factor grouped items 4-7, 9, 10, 11, 13, 15-18, 22. Item 7 (“I watch videos on how to speak well”; factor loading = 0.75) was the most representative of this factor. The threat factor comprised items 26-31, 33-35, 37-39, 42-44, 46 and 47. Item 30 (“I make excuses not to speak in public”; factor loading = 0.86) was the most representative.

**Table 4:** Factorial structure of ECOFAP

Items	Factor 1 (Challenge)	Factor 2 (Threat)
4- I strive to pronounce my words clearly.	0.32	
5- I read about how to speak well in public.	0.74	
6- I observe how good speakers speak.	0.48	
7- I watch videos on how to speak well.	0.75	
9- I visualize myself speaking well.	0.38	
10- I try to maintain a confident body posture.	0.32	
12- I try to remain calm while I speak.	0.40	
13- I seek professional support to learn how to speak well in public.	0.55	
15- I practice my speech with people I know.	0.38	
16- I seek suggestions from colleagues on how they handle the situation.	0.47	
17- I listen to music to relax before speaking in public.	0.31	
18- I take walks as a distraction before a public presentation.	0.33	
22- I negotiate more time to prepare myself.	0.36	
26- I see myself paralyzed when speaking in public.		0.80
27- I try to speak better, but I can't.		0.77
28- I think about what I'm going to say, but I can't find a way out.		0.79
29- I postpone public speaking.		0.84
30- I make excuses not to speak in public.		0.86
31- I speed up my speech to finish quickly.		0.68
33- I complain to colleagues about having to be the one to speak.		0.75
34- I blame myself for not wanting to speak in public.		0.82
35- I ask someone else to speak in my place.		0.83
37- I avoid talking to my colleagues about my difficulty.		0.53
38- I hide my feelings from my colleagues when I have to speak.		0.54
39- I avoid places where I have to talk.		0.80
42- I insist on the idea that I will not succeed.		0.78
43- I keep telling myself that it's hard to cope with this situation.		0.80
44- I tell myself that my presentation will go wrong.		0.71
46- I get irritated when someone disagrees with what I'm saying.		0.31
47- I get impatient when others ask me to repeat what I said.		0.39
Total Cronbach's alpha = 0.89		
Total variance explained = 78%		

**Figure 1:**
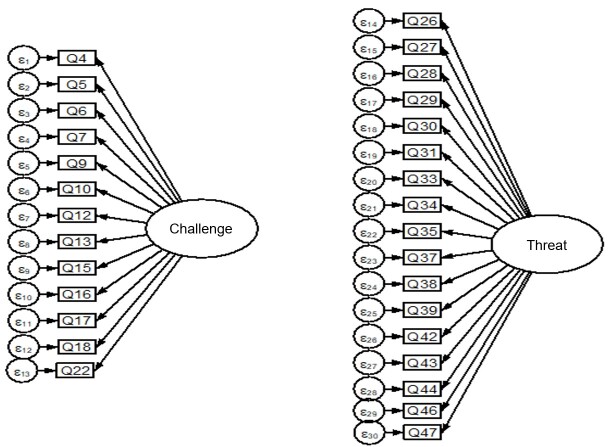
ECOFAP factorial structure diagram

Figure 1 presents the path diagram for the ECOFAP two-factor structure.

The final version of the ECOFAP had a two-factor structure with 30 items and Likert-type response categories from 1 (strongly disagree) to 5 (strongly agree) (Appendix A). The items were randomized in descending order according to their factor loading, eigenvalues, and domain variance.

## DISCUSSION

The results demonstrated validity based on the internal structure of the ECOFAP, measured with a sample of Brazilian adults who speak publicly in academic or professional contexts. ECOFAP followed rigorous methodological criteria recommended by the SEPT^(1)^ manual and is a self-assessment tool for coping strategies appropriate for public speaking. Self-assessment helps people question a specific aspect, which is often not spontaneously reported in interventions, and influences their readiness to change their problem^(20,21)^. This self-assessment verified their perceptions of their ability to self-regulate their behaviors.

An instrument's reliability is measured through internal consistency, which determines whether all items measure the same construct. The ECOFAP presented satisfactory internal consistency values, measured by Cronbach's alpha, across all items, indicating that the instrument meets the values ​​recommended in the literature^(22,23)^ and that all items adequately represent the construct of coping with public speaking.

Factor structure analysis (dimensionality) verifies whether the relationships between the instrument's items are aligned with the theoretical framework proposed for the construct. The psychometric results of the ECOFAP revealed a two-factor structure (challenge and threat). Validation studies of measurement instruments that used the CMT to assess coping in other stressful situations also failed to categorize their instruments into 12 coping dimensions. They found two dimensions categorized into adaptive and maladaptive coping families^(24-26)^. Therefore, the results obtained from the analysis of the instrument's factor structure are consistent with the findings in the literature on coping based on the theoretical model in question.

Based on the factor structure obtained for the ECOFAP, the authors propose that the scoring and interpretation of scores consider individuals' coping strategies, organized into two factors: challenge and threat. The challenge factor includes behaviors that help positively cope with anxiety when speaking in public, such as practicing speaking and enunciating words clearly, which improve self-perception and communication. The threat factor, on the other hand, involves behaviors that avoid exposure to public speaking, such as speeding up speech or making excuses (i.e., strategies that may provide immediate relief but are considered dysfunctional). In ECOFAP, the challenge factor comprises items 1, 3, 5, 7, 10, 11, 15, 18, 20, 22, 25, 28 and 30. The threat factor is formed by items 2, 4, 6, 8, 9, 12, 13, 14, 16, 17, 19, 21, 23, 24, 26, 27 and 29.

By simply adding up the items in their respective domains, it is possible to determine which coping factor is most prevalent. The total challenge factor score can range from 13 to 65 points, while the total threat factor score can range from 17 to 85 points. The higher the total challenge factor score, the better the coping with public speaking. These quantitative scores can also be useful for investigating associations between the two domains and different public speaking outcomes. The items can also be analyzed qualitatively, observing which families received the most responses. This approach allows for targeted interventions aligned with the predominant coping profile identified. The Appendix presents the ECOFAP and a table with the items corresponding to each family.

We believe that ECOFAP can help individuals who speak in public understand their coping strategies. The scale will help to develop more effective and personalized interventions and counseling to improve oral communication and reduce the impact of anxiety and stress during presentations.

The limitations of this study are related to the lack of a gold standard instrument for assessing coping strategies for public speaking, which precludes further comparative analyses. We suggest that future studies evaluate the scale's relationships with other coping measures, seeking to assess convergent and divergent validity, as well as test-retest validity to assess the instrument's stability.

## CONCLUSION

The ECOFAP consists of 30 items. The scale allows for a comprehensive assessment of coping strategies for public speaking. Factor and internal consistency analyses show that the ECOFAP is a reliable and valid instrument for assessing coping strategies related to public speaking.

## Appendix A Speaking in Public Coping Scale – ECOFAP

This scale aims to assess the strategies you currently use to deal with public speaking situations. Consider “public speaking” as communicating loudly in front of strangers in a professional or academic context.

For each statement below, mark from 1 to 5 how much you agree or disagree with the statement, with 1 meaning “strongly disagree”, and 5 meaning “strongly agree.”

**Table:**

1. I read about how to speak well in public	1	2	3	4	5
2. I make excuses not to speak in public	1	2	3	4	5
3. I struggle to pronounce the words clearly	1	2	3	4	5
4. I postpone the situation of speaking in public	1	2	3	4	5
5. I seek professional support to learn how to speak well in public.	1	2	3	4	5
6. I blame myself for not wanting to speak in public	1	2	3	4	5
7. I visualize myself speaking well	1	2	3	4	5
8. I ask someone else to speak in my place.	1	2	3	4	5
9. I get impatient when others ask me to repeat what I said.	1	2	3	4	5
10. I negotiate more time to prepare myself	1	2	3	4	5
11. I observe how good speakers speak	1	2	3	4	5
12. I see myself paralyzed when speaking in public	1	2	3	4	5
13. I avoid talking to my colleagues about my difficulty	1	2	3	4	5
14. I avoid places where I have to talk	1	2	3	4	5
15. I seek suggestions from colleagues on how they handle the situation	1	2	3	4	5
16. I keep telling myself that it's hard to cope with this situation	1	2	3	4	5
17. I hide my feelings from my colleagues when I have to speak	1	2	3	4	5
18. I try to maintain a confident body posture	1	2	3	4	5
19. I reflect on what I'm going to say, but I can't find a way out	1	2	3	4	5
20. I practice my speech with people I know	1	2	3	4	5
21. I insist on the idea that I will not succeed	1	2	3	4	5
22. I try to remain calm while I speak	1	2	3	4	5
23. I try to speak better, but I can't	1	2	3	4	5
24. I complain to colleagues about having to be the one to speak	1	2	3	4	5
25. I take walks as a distraction before a public presentation	1	2	3	4	5
26. I tell myself my presentation will go wrong	1	2	3	4	5
27. I speed up my speech to finish quickly	1	2	3	4	5
28. I listen to music to relax before speaking in public	1	2	3	4	5
29. I get irritated when someone disagrees with what I'm saying.	1	2	3	4	5
30. I watch videos on how to speak well	1	2	3	4	5

Coping families and ECOFAP items

**Table:**

Coping families	Items
**CHALLENGE FACTOR**	
Seeking information: actively trying to learn more about the situation. Reading, studying, asking others.	1,11, 30
Solving problems: actively trying to change the situation or its consequences. Planning strategies, instrumental actions.	3
Self-confidence: actively trying to reduce emotional distress. Behavioral regulation and positive self-talk.	7, 18, 22
Adapting: Actively trying to redirect attention away from the stressful situation, engaging in pleasurable activities, or changing one's perspective on the situation. Cognitive restructuring, acceptance, minimization.	25, 28
Seeking support: actively trying to use available social resources to deal with stressful situations through seeking contact, help, and social referencing.	5, 15, 20
Negotiating: actively trying to find new options, balancing individual priorities with the constraints of the situation, and establishing new goals. Exchanging.	10
**THREAT FACTOR**	
Helplessness: the individual considers themselves powerless in the face of the situation. Passivity, doubt, discouragement	12, 23, 19
Delegation: the individual feels sorry for themselves when they believe they do not have sufficient resources to deal with the stressful event. Complaining, self-blaming, regretting.	6, 8, 24
Avoidance: actively trying to free themselves from the situation and its related difficulties. Pessimism, mental withdrawal.	2, 4, 27
Isolation: Actively trying to avoid people or prevent them from knowing how you feel about the situation. Social withdrawal, avoidance of others, loneliness.	13,14, 17
Submission: actively trying to maintain passive and repetitive focus on the negative and harmful aspects of the situation. Rigidity, rumination, intrusive thoughts.	16, 21, 26
Opposition: Actively trying to use angry, explosive behaviors to remove obstacles imposed by the stressful situation. Aggression, explosiveness, blaming others.	9, 29

**ECOFAP correction:** The simple sum of each factor is calculated:

**Challenge:** items 1, 3, 5, 7, 10, 11, 15, 18, 20, 22, 25, 28 and 30 (13 items; maximum score: 65)

**Threat:** items 2, 4, 6, 8, 9, 12, 13, 14, 16, 17, 19, 21, 23, 24, 26, 27 and 29 (17 items; maximum score: 85)
