# Supplementary material for: Speaking in Public Coping Scale (ECOFAP): validity evidence based on internal structure
Source: Codas. 2025 Dec 8;37(6):e20240309. doi: 10.1590/2317-1782/e20240309en (PMC12704609; doi:10.1590/2317-1782/e20240309en)
Supplement: Translation

# Translation: Escala de *Coping* para Fala em Público (ECOFAP): evidências de validade baseadas na estrutura interna

**DOI:** 10.1590/2317-1782/e20240309pt

**Authors:** Anna Carolina Ferreira Marinho, Adriane Mesquita de Medeiros, Eduardo de Paula Lima, Letícia Caldas Teixeira

**Affiliations:** 1 Programa de Pós-graduação em Ciências Fonoaudiológicas, Universidade Federal de Minas Gerais – UFMG - Belo Horizonte (MG), Brasil.; 2 Departamento de Fonoaudiologia, Faculdade de Medicina, Universidade Federal de Minas Gerais – UFMG - Belo Horizonte (MG), Brasil.; 3 Corpo de Bombeiros Militar de Minas Gerais - Belo Horizonte (MG), Brasil.

**Keywords:** Estudo de Validação, Coping, Fala em Público, Autoavaliação, Psicometria

## Abstract

**Objetivo:**

Apresentar as evidências de validade da Escala de Coping para Fala em Público (ECOFAP) baseadas na estrutura interna.

**Método:**

Estudo metodológico de elaboração e validação de instrumento. Participaram do estudo 1.119 adultos que falam em público em contextos acadêmicos ou profissionais, com média de 25 anos. A maioria da amostra era do sexo feminino (68,5%) e com ensino superior completo (58,5%). Foram utilizados para a validação um questionário de autorrelato incluindo: 1) características sociodemográficas (idade, sexo, escolaridade, profissão); 2) perguntas sobre participação em treinamentos para aperfeiçoamento da comunicação em público e experiência de fala em público, em contextos acadêmicos e profissionais; 3) Escala de Coping para Fala em Público (ECOFAP de 48 itens). Para as etapas de evidência de validade, foram realizadas análises da consistência interna (alfa de Cronbach), Análise Fatorial Exploratória (AFE) e Análise Fatorial Confirmatória (AFC).

**Resultados:**

Após a conclusão das análises, a ECOFAP ficou composta por 30 itens. Os resultados indicaram uma boa consistência interna da ECOFAP (alfa de Cronbach 0,87). O instrumento apresentou estrutura fatorial composta pelo fator Desafio (13 itens) e Ameaça (17 itens). As cargas fatoriais variaram de 0,37 a 0,66.

**Conclusão:**

A ECOFAP mostra uma estrutura interna sólida e propriedades psicométricas adequadas. O instrumento é confiável e válido para autoavaliar estratégias de coping para fala em público de indivíduos adultos em contextos acadêmicos e profissionais.

## INTRODUÇÃO

A Escala de *Coping* para Fala em Público (ECOFAP) é um instrumento de autoavaliação destinado a avaliar as estratégias de autorregulação de adultos durante o processo da fala em público. A sua elaboração seguiu os critérios estabelecidos pelo manual *Standards for Educational and Psychological Testing-SEPT*, que define as etapas metodológicas para o desenvolvimento e validação de instrumentos de medida. Essas etapas incluem a obtenção de evidências de validade baseadas no conteúdo do teste, nos processos de resposta, na estrutura interna, na relação com outras variáveis e nas consequências dos testes^(1)^.

A construção da ECOFAP foi fundamentada nos referenciais teóricos da fala em público^(2)^ e na Teoria Motivacional do *Coping* (TMC)^(3-5)^. Compreende-se a fala em público como a habilidade de transmitir oralmente uma mensagem para uma plateia em diversos contextos, sejam eles acadêmicos, sociais ou profissionais^(6,7)^. Nessa atividade comunicativa, o indivíduo enfrenta desafios, como desenvolver um discurso coerente, informar e entreter o público, adaptar a mensagem ao público-alvo, apresentar-se de forma clara e confiante, além de lidar com o nervosismo e o medo da exposição^(7-11)^.

A TMC é uma proposta desenvolvimentista de análise do enfrentamento do estresse, que se baseia na abordagem do *coping* como uma ação regulatória dos próprios padrões de comportamentos, motivações e emoções diante de condições estressantes^(3-5)^. O evento estressante é percebido como um desafio ou uma ameaça às necessidades psicológicas básicas de relacionamento, competência e autonomia^(3-5,12,13)^. A teoria descreve 12 estratégias/famílias de enfrentamento, segundo um desfecho adaptativo positivo (desafio): autoconfiança, busca de suporte, resolução de problemas, busca de informações, acomodação e negociação; ou um desfecho negativo (ameaça): delegação, isolamento, desamparo, fuga, submissão e oposição^(3-5)^.

Acredita-se que a conscientização pessoal das estratégias de enfrentamento na fala em público pode auxiliar as pessoas a lidar melhor com situações comunicativas. Esse conhecimento pode contribuir para o desenvolvimento de intervenções e assessorias específicas voltadas a aprimorar a comunicação e promover o bem-estar emocional e mental diante das atividades de fala em público.

A validação da ECOFAP foi realizada em duas etapas segundo as diretrizes de validação de instrumentos^(1)^. A primeira, denominada validação do conteúdo e do processo de resposta, já publicada em artigo anterior, demonstrou que os itens possuem estrutura semântica e sintática adequadas para representar o construto de fala em público e *coping* ^(14)^. Neste artigo, seguindo as recomendações do SEPT^(1)^, apresenta-se a segunda etapa: validação da estrutura interna, que avalia a consistência interna, a validade e a confiabilidade das medidas. Com base nessas considerações, o objetivo deste estudo é apresentar as evidências de validade da ECOFAP baseadas na estrutura interna.

## MÉTODO

### Tipo de estudo

Estudo metodológico de elaboração e validação de instrumento de medida, aprovado pelo Comitê de Ética em Pesquisa (CEP), parecer 5.735.670/2022.

### Participantes

Foram convidados a participar do estudo indivíduos adultos de ambos os sexos. Os seguintes critérios de inclusão foram estabelecidos: ter vivenciado situações de fala em público nos contextos acadêmicos e ocupacionais e ter pelo menos 18 anos de idade. Foram excluídos da amostra estudantes e profissionais de Psicologia e Fonoaudiologia, bem como aqueles que já haviam participado de qualquer aperfeiçoamento em comunicação oral em público, evitando viés de seleção. A seleção dos participantes foi realizada por meio de uma amostra não probabilística, utilizando uma abordagem de amostragem tipo bola de neve. Inicialmente, 1.190 indivíduos responderam à pesquisa. Destes, 71 participantes foram excluídos devido aos critérios de elegibilidade estabelecidos. A amostra final foi composta por 1.119 adultos, incluindo 308 estudantes universitários, 250 advogados, 253 empreendedores, 100 professores universitários, 100 executivos, 61 palestrantes e 46 políticos.

### Recrutamento dos participantes

A pesquisa foi divulgada em diversos ambientes virtuais, como grupos de fonoaudiólogos especializados em aperfeiçoamento da comunicação, perfis no Instagram, e-mails institucionais de uma universidade pública brasileira, setores de recursos humanos de empresas de tecnologia, escritórios de advocacia e gabinetes políticos. A coleta de dados foi realizada ao longo de quatro meses por meio da plataforma Google *Forms.* Os participantes receberam uma carta convite contendo o link do Termo de Consentimento Livre e Esclarecido (TCLE), e um questionário com o instrumento de avaliação.

### Instrumento para validação

O questionário continha perguntas para descrição de características sociodemográficas (idade, sexo, escolaridade, profissão), uma pergunta sobre participação em treinamentos para aperfeiçoamento da comunicação e uma pergunta sobre experiência de falar em público em contextos profissionais ou acadêmicos. O questionário incluiu ainda a ECOFAP. A chave de resposta da escala era do tipo *Likert* variando de 1 (discordo totalmente) a 5 (concordo totalmente) pontos. A ECOFAP piloto foi composta por 48 itens, incluindo as 12 categorias/famílias de *coping*: “resolução de problemas”, “busca de informações”, “desamparo”, “fuga”, “autoconfiança”, “busca de apoio”, “delegação”, “isolamento”, “acomodação”, “negociação”, “submissão”, “oposição”. Os itens foram dispostos aleatoriamente para os participantes e sem a indicação das respectivas famílias que eles representavam.

### Análise de dados

Realizou-se análise descritiva das variáveis sociodemográficas. Posteriormente foram conduzidas análises das propriedades psicométricas da ECOFAP, medidas de confiabilidade e dimensionalidade, utilizando os seguintes testes: o coeficiente de alfa de *Cronbach*, Análise Fatorial Exploratória (AFE) e Análise Fatorial Confirmatória (AFC).

O coeficiente alfa de *Cronbach* foi calculado para avaliar a consistência interna da ECOFAP, considerando valores >0,60 como indicativos de boa confiabilidade^(15-18)^. A viabilidade da AFE foi verificada pelo Índice de *Kaiser-Meyer-Olkin* (KMO), sendo considerados adequados valores >0,70 e pelo Teste de Esfericidade de *Bartlett*, com p-valor <0,05. A AFE seguiu a análise dos componentes principais, adotando o critério de Kaiser, que mantém fatores com autovalores >1. As cargas fatoriais foram extraídas pelo método de rotação ortogonal *Varimax*, considerando como adequados valores >0,30. A análise foi realizada no software *Statistical Package for the Social Sciences - SPSS, versão 26.*

A AFE foi realizada em duas etapas. Na etapa inicial foram avaliados 48 itens, quatro em cada família de *coping*. Com o objetivo de reduzir o instrumento de medida e seguindo o pressuposto teórico de manter três itens por família de *coping*, os autores retiraram um item de cada domínio da ECOFAP. Os critérios utilizados para a retirada foram: itens com estrutura semântica e cargas fatoriais semelhantes dentro do mesmo domínio e itens com cargas fatoriais abaixo dos valores recomendados pela literatura. Com base nos resultados da AFE a AFC foi então realizada por meio do programa de Modelagem de Equação Estrutural (MEE).

Para ajuste do modelo, foram considerados os seguintes critérios: Qui-quadrado normado x^2^/gl (valores entre 1 e 5); índice de normalidade de ajuste GFI (>0,90); índice de qualidade de ajuste ajustado AGFI (>0,90); raiz média quadrática dos erros de aproximação MSEA (0,05-0,10); índice *Tukey-Lewis* TLI (>0,90); índice de ajuste comparativo CFI (>0,90)^(18,19)^. Essa análise foi realizada no software *Stata Corp, College Station*-STATA versão 12.

## RESULTADOS

A faixa etária dos participantes variou entre 18 e 66 anos, com uma média de 22 anos. A maioria era do sexo feminino (n = 697, 68,5%) e possuía ensino superior completo (n = 596, 58,5%). A análise dos componentes principais (autovalores) e a variância dos 12 fatores da ECOFAP estão representados na Tabela 1. Os autovalores variaram de 1,60 (resolução de problemas) a 3,01 (fuga) e a variância de 53,42 (resolução de problemas) a 89,49 (submissão).

**Tabela 1:** Análise dos componentes principais da ECOFAP

Fatores da ECOFAP	Autovalores	% variância
Resolução de problemas	1,60	53,42
Busca de informações	1,95	65,1211
Autoconfiança	1,98	66,11
Busca de apoio	2,24	55,96
Acomodação	1,70	56,74
Negociação	1,80	60,25
Desamparo	2,43	81,17
Fuga	2,39	79,76
Delegação	3,01	75,33
Isolamento	2,61	65,29
Submissão	2,47	89,49
Oposição	2,12	70,79

A estrutura interna da ECOFAP, proposta na AFE encontra-se na Tabela 2. O processo foi realizado em duas etapas. Na primeira etapa, a ECOFAP, versão piloto de 48 itens, foi utilizada para análise inicial. Os coeficientes de correlação item-total variaram de 0,08 a 0,76. Os valores de alfa de *Cronbach* variaram de 0,57 a 0,90 e as cargas fatoriais variaram de 0,14 a 0,66. Seguindo o pressuposto teórico da TMC de manter três itens por família de *coping*, 12 itens foram retirados, segundo os respectivos valores dos coeficientes de correlação item-total, alfa de *Cronbach* e as cargas fatoriais. Dessa análise 7 itens apresentaram baixos coeficientes de correlação item-total (itens 3,8,14,20,21,25,40), 1 (item 21) apresentou carga fatorial não significativa, 5 (itens 11,32,36,41,48) excluídos aumentaram os valores de alfa de *Cronbach* dos seus respectivos domínios, além de obterem menores cargas fatorais dentro do mesmo domínio. Após a exclusão dos 12 itens, foi realizada uma nova análise da ECOFAP com os 36 itens. Nessa segunda etapa, todos os parâmetros foram estatisticamente significativos. Os coeficientes de correlação item-total variaram de 0,30 a 0,090, alfa de *Cronbach* variaram de 0,60 a 0,89, e as cargas fatoriais com pesos variando de 0,37 a 0,66.

**Tabela 2:** Análise Fatorial Exploratória do instrumento ECOFAP: etapa 1 versão ECOFAP- 48 itens e etapa 2 versão ECOFAP-36 itens

Domínios e Itens	ETAPA 1- ECOFAP 48 itens			ETAPA 2- ECOFAP 36 itens		
	Correlação item-total	*a* se o item for excluído	Carga Fatorial	Correlação item-total	*a* se o item for excluído	Carga fatorial
**Resolução de problemas**		0,59			0,60	
**1**	0,50	0,45	0,57	0,51	0,60	0,63
**2**	0,31	0,51	0,53	0,30	0,44	0,61
3 ***	0,20	0,60	0,48	-	-	-
**4**	0,50	0,58	0,39	0,49	0,55	0,46
**Busca de informações**		0,73			0,72	
**5**	0,30	0,65	0,52	0,31	0,53	0,62
**6**	0,30	0,69	0,48	0,32	0,82	0,45
**7**	0,30	0,62	0,54	0,30	0,65	0,63
8 ***	0,21	0,72	0,44	-	-	-
**Autoconfiança**		0,75			0,76	
**9**	0,70	0,72	0,46	0,90	0,75	0,52
**10**	0,34	0,65	0,54	0,33	0,66	0,61
11***	0,37	0,76	0,46	-	-	-
**12**	0,70	0,68	0,52	0,70	0,68	0,52
**Busca de apoio**		0,72			0,60	
**13**	0,31	0,75	0,36	0,31	0,60	0,61
14 ***	0,26	0,60	0,57	-	-	-
**15**	0,32	0,72	0,44	0,32	0,43	0,48
**16**	0,32	0,56	0,58	0,31	0,31	0,59
**Acomodação**		0,59			0,60	
**17**	0,34	0,43	0,58	0,34	0,39	0,63
**18**	0,44	0,45	0,57	0,43	0,44	0,62
**19**	0,30	0,57	0,42	0,30	0,65	0,61
20 ***	0,08	0,60	0,38	-	-	-
**Negociação**		0,57			0,65	
**21** ^***^	0,17	0,65	0,14	-	-	-
**22**	0,33	0,50	0,37	0,31	0,80	0,47
**23**	0,64	0,36	0,66	0,64	0,34	0,37
**24**	0,72	0,39	0,65	0,72	0,39	0,66
**Desamparo**		0,90			0,88	
25 ***	0,22	0,88	0,49	-	-	-
**26**	0,72	0,87	0,50	0,73	0,87	0,55
**27**	0,65	0,87	0,50	0,64	0,80	0,58
**28**	0,69	0,87	0,50	0,69	0,80	0,58
**Fuga**		0,90			0,84	
**29**	0,73	0,85	0,51	0,73	0,72	0,60
**30**	0,73	0,85	0,52	0,73	0,71	0,60
**31**	0,62	0,92	0,42	0,62	0,89	0,52
32 ***	0,76	0,84	0,52	-	-	-
**Delegação**		0,88			0,86	
**33**	0,67	0,86	0,49	0,66	0,80	0,58
**34**	0,71	0,84	0,50	0,71	0,87	0,55
**35**	0,74	0,84	0,51	0,74	0,76	0,59
36 ***	0,69	0,86	0,48	-	-	-
**Isolamento**		0,82			0,79	
**37**	0,46	0,77	0,50	0,46	0,66	0,60
**38**	0,48	0,76	0,51	0,48	0,62	0,61
**39**	0,67	0,77	0,49	0,67	0,85	0,50
40 ***	0,25	0,79	0,47	-	-	-
**Submissão**		0,89			0,89	
41***	0,59	0,89	0,47	-	-	-
**42**	0,67	0,84	0,52	0,65	0,82	0,58
**43**	0,69	0,87	0,50	0,69	0,85	0,57
**44**	0,60	0,86	0,49	0,61	0,85	0,57
**Oposição**		0,77			0,79	
**45**	0,32	0,71	0,50	0,33	0,75	0,56
**46**	0,32	0,68	0,53	0,34	0,67	0,59
**47**	0,40	0,68	0,53	0,41	0,71	0,57
48 ***	0,46	0,79	0,40	-	-	-

*** item excluído do instrumento; ***a*** coeficiente de alfa de *Cronbach*

A Tabela 3 apresenta os índices de ajustes do MEE. Os valores iniciais dos índices GFI, AGFI, RMSEA, TLI, CFI e PGFI estão abaixo do mínimo preconizado na literatura^(19)^, não podendo confirmar a estrutura fatorial da ECOFAP com 12 fatores. Os autores decidiram testar uma nova estrutura de dois fatores, agrupando seis famílias de *coping* com foco no desafio (resolução de problemas, busca de informações, autoconfiança, busca de apoio, acomodação e negociação) e seis com foco na ameaça (desamparo, fuga, delegação, isolamento, submissão e oposição). Destaca-se ainda que, conforme exposto, os valores se apresentaram adequados. Nesse processo foi necessário excluir os itens 24(0,11), 23(0,13), 1(0,15), 19(0,19), 45(0,28) e 2(0,29), começando pelo menor valor pois as cargas fatoriais foram abaixo do nível de significância de 0,30.

**Tabela 3:** Indicadores de ajuste da Modelagem de Equação Estrutural para validação da ECOFAP

Indicadores de ajuste	Modelo Final
**ECOFAP-12 Fatores**	
Função de discrepância: x^2^ (valor p)	29.380
Qui-quadrado normado (x^2^/gl)	0,59
GFI (índice de normalidade de ajuste)	0,78
AGFI (índice de qualidade de ajuste ajustado)	0,73
RMSEA (raiz média quadrática dos erros de aproximação)	0,50
TLI (índice Tukey-Lewis)	0,85
CFI (Índice de ajuste comparativo)	0,78
**ECOFAP- 2 Fatores**	
Função de discrepância: x^2^ (valor p)	4392.291
Qui-quadrado normado (x^2^/gl)	1,73
GFI (índice de normalidade de ajuste)	0,96
AGFI (índice de qualidade de ajuste ajustado)	0,93
RMSEA (raiz média quadrática dos erros de aproximação)	0,05
TLI (índice Tukey-Lewis)	0,96
CFI (Índice de ajuste comparativo)	0,92

A estrutura multifatorial considerando dois fatores para a ECOFAP está apresentada na Tabela 4. Verificou-se que o Fator Desafio agrupou os itens 4-7,9,10,11,13,15-18,22, sendo o item 7 “*Assisto vídeos sobre como falar bem.”* (carga fatorial = 0,75) o mais representativo deste fator. O Fator Ameaça foi composto pelos itens (26-31,33-35,37-39,42-44, 46 e 47), sendo o item 30 *“Invento uma desculpa para não falar em púbico”* (carga fatorial = 0,86) o mais representativo.

**Tabela 4:** Estrutura fatorial da ECOFAP

Itens	Fator 1 (Desafio)	Fator 2 (Ameaça)
4- Me esforço para pronunciar as palavras com clareza.	0,32	
5- Leio sobre como falar bem em público.	0,74	
6- Observo como bons falantes falam.	0,48	
7- Assisto vídeos sobre como falar bem.	0,75	
9- Visualizo-me falando bem.	0,38	
10- Tento manter uma postura corporal confiante.	0,32	
12- Procuro manter-me tranquilo enquanto falo.	0,40	
13- Busco apoio de um profissional para aprender a falar bem em público.	0,55	
15- Treino a minha fala com pessoas conhecidas.	0,38	
16- Procuro sugestões de como os colegas lidam com a situação.	0,47	
17- Escuto música para relaxar antes de falar em público.	0,31	
18- Faço caminhadas para me distrair antes de uma apresentação em público.	0,33	
22- Negocio um prazo maior para me preparar.	0,36	
26- Me imagino travado na hora de falar em público.		0,80
27- Tento falar melhor, mas não consigo.		0,77
28- Reflito sobre o que vou falar, mas não encontro uma saída.		0,79
29- Adio a situação de falar em público.		0,84
30- Invento uma desculpa para não falar em público.		0,86
31- Acelero a fala para terminar logo.		0,68
33- Reclamo com colegas por ter que ser eu a falar.		0,75
34- Me culpo por não querer falar em público.		0,82
35- Peço que alguém fale em meu lugar.		0,83
37- Evito conversar com meus colegas sobre minha dificuldade.		0,53
38- Escondo dos colegas o que sinto quando sou obrigado a falar.		0,54
39- Evito lugares onde tenha que falar.		0,80
42- Insisto na ideia de que não terei sucesso.		0,78
43- Repito para mim mesmo que é difícil enfrentar essa situação.		0,80
44- Digo a mim mesmo que a minha apresentação vai dar errado.		0,71
46- Demonstro irritação quando alguém discorda do que eu estou falando.		0,31
47- Reajo com impaciência quando os outros pedem para repetir o que falei.		0,39
Alfa de *Cronbach* total =0,89		
Variância total explicada =78%		

A Figura 1 apresenta o diagrama de caminhos para a estrutura de dois fatores da ECOFAP.

**Figura 1:** Diagrama estrutura fatorial ECOFAP

A versão final da ECOFAP apresentou uma estrutura de dois fatores com 30 itens e categorias de resposta do tipo *likert 1(discordo totalmente) a 5 (concordo totalmente)* (Apêndice A). Os itens foram aleatorizados em ordem decrescente de acordo com sua carga fatorial, autovalores e variância dos domínios.

## DISCUSSÃO

Os resultados mostraram as evidências de validade baseada na estrutura interna da ECOFAP, medida com uma amostra de indivíduos adultos brasileiros que falam em público em contextos acadêmicos ou profissionais. A ECOFAP seguiu rigorosos critérios metodológicos recomendado pelo manual SEPT^(1)^, sendo um instrumento de autoavaliação de estratégias de *coping* adequado para falar em público. A autoavaliação auxilia o sujeito a se questionar sobre determinado aspecto, que muitas vezes não é relatado espontaneamente em intervenções, e influencia na prontidão para mudança em relação ao seu problema^(20,21)^. A partir da autoavaliação, verifica-se a percepção que a pessoa tem sobre a sua capacidade de autorregulação em relação aos seus comportamentos.

A análise de confiabilidade de um instrumento é mensurada por meio da consistência interna, usada para determinar se todos os itens de um instrumento medem o mesmo construto. A ECOFAP apresentou valores satisfatórios de consistência interna, medidos pelo coeficiente Alfa de *Cronbach*, em todos os itens, o que indica que o instrumento atende aos valores recomendados na literatura^(22,23)^ e que todos os itens representam adequadamente o construto do *coping* para falar em público.

A análise da estrutura fatorial (dimensionalidade) verifica se as relações entre os itens do instrumento estão alinhadas com a estrutura teórica proposta para o construto. Os resultados psicométricos da ECOFAP mostraram uma estrutura de dois fatores (desafio e ameaça). Estudos de validação de instrumentos de medida que utilizaram a TMC para avaliar o *coping* em outras situações estressoras também não conseguiram categorizar seus instrumentos em 12 dimensões de enfrentamento. Eles encontraram duas dimensões categorizadas em famílias adaptativas e mal adaptativas de enfrentamento^(24-26)^. Nesse sentido, os resultados obtidos para a análise da estrutura fatorial do instrumento são condizentes com os achados da literatura sobre *coping* a partir do modelo teórico em foco.

A partir da estrutura fatorial obtida para a ECOFAP, os autores propõem que a cotação e a interpretação dos escores considere as estratégias de enfrentamento dos indivíduos organizadas em dois fatores: desafio e ameaça. O fator “desafio” inclui comportamentos que ajudam a enfrentar positivamente a ansiedade ao falar em público, como praticar a fala e pronunciar as palavras com clareza, o que melhora a autopercepção e a comunicação. O fator “ameaça”, por outro lado, envolve comportamentos que evitam a exposição à fala em público, como acelerar a fala ou inventar desculpas, estratégias que podem proporcionar alívio imediato, mas são consideradas disfuncionais. Na ECOFAP, o fator “desafio” é composto pelos itens 1, 3, 5, 7, 10, 11, 15, 18, 20, 22, 25, 28 e 30. Já o fator “ameaça” é formado pelos itens 2, 4, 6, 8, 9, 12, 13, 14, 16, 17, 19, 21, 23, 24, 26, 27 e 29.

Por meio da soma simples dos itens em seus respectivos domínios é possível determinar qual dos fatores de enfrentamento é mais predominante. A pontuação total do fator desafio pode variar de 13 a 65 pontos, enquanto a pontuação total do fator ameaça pode variar de 17 a 85 pontos. Quanto maior for a pontuação total do fator desafio, melhor será o enfrentamento da fala em público. Esses escores quantitativos também podem ser úteis para investigar associações entre os dois domínios e diferentes desfechos de fala em público. Os itens também podem ser analisados qualitativamente observando-se quais famílias foram mais assinaladas. Tal abordagem possibilita o direcionamento de intervenções alinhadas ao perfil predominante de enfrentamento identificado. No Apêndice apresenta-se a ECOFAP e um quadro com os itens correspondentes a cada família.

Acreditamos que a ECOFAP pode auxiliar indivíduos que falam em público a compreender suas estratégias de enfrentamento. A escala contribuirá para o desenvolvimento de intervenções ou assessorias mais eficazes e personalizadas, com o objetivo de aprimorar a comunicação oral e reduzir o impacto da ansiedade e do estresse nas apresentações.

As limitações deste estudo estão relacionadas à ausência de um instrumento padrão-ouro de avaliação de estratégias de *coping* para falar em público, impossibilitando a realização de outras análises comparativas. Sugerimos que futuros estudos possam avaliar as relações da escala com outras medidas relacionadas ao *coping*, buscando avaliar evidências de validade convergente e divergente, bem como teste-reteste para avaliar a estabilidade do instrumento.

## CONCLUSÃO

A ECOFAP ficou composta por 30 itens. A escala permite uma avaliação abrangente das estratégias de enfrentamento para fala em público. As análises fatoriais e de consistência interna mostram que a ECOFAP é um instrumento confiável e válido para avaliar estratégias de *coping* relacionadas à fala em público.

## Apêndice A Escala *Coping* para fala em público - ECOFAP

Esta escala tem o objetivo de avaliar quais são as estratégias que você usa, no momento atual da sua vida, para enfrentar situações de fala em público. Considere “falar em público” como se comunicar em voz alta, diante de pessoas desconhecidas, em um contexto profissional ou acadêmico.

Diante de cada afirmação abaixo, assinale de 1 a 5 o quanto você concorda ou discorda da afirmação, sendo 1 para “discordo totalmente” e 5 para “concordo totalmente”.

**Table:**

1.Leio sobre como falar bem em público	1	2	3	4	5
2.Invento uma desculpa para não falar em público	1	2	3	4	5
3. Me esforço para pronunciar as palavras com clareza	1	2	3	4	5
4. Adio a situação de falar em público	1	2	3	4	5
5. Busco apoio de um profissional para aprender a falar bem em público	1	2	3	4	5
6. Me culpo por não querer falar em público	1	2	3	4	5
7.Visualizo-me falando bem	1	2	3	4	5
8. Peço que alguém fale em meu lugar	1	2	3	4	5
9. Reajo com impaciência quando os outros pedem para repetir o que falei	1	2	3	4	5
10. Negocio um prazo maior para me preparar	1	2	3	4	5
11.Observo como bons falantes falam	1	2	3	4	5
12. Me imagino travado na hora de falar em público	1	2	3	4	5
13. Evito conversar com meus colegas sobre minha dificuldade	1	2	3	4	5
14. Evito lugares onde tenha que falar	1	2	3	4	5
15. Procuro sugestões de como os colegas lidam com a situação	1	2	3	4	5
16. Repito para mim mesmo que é difícil enfrentar essa situação	1	2	3	4	5
17. Escondo dos colegas o que sinto quando sou obrigado a falar	1	2	3	4	5
18. Tento manter uma postura corporal confiante	1	2	3	4	5
19. Reflito sobre o que vou falar, mas não encontro uma saída	1	2	3	4	5
20. Treino a minha fala com pessoas conhecidas	1	2	3	4	5
21. Insisto na ideia de que não terei sucesso	1	2	3	4	5
22. Procuro manter-me tranquilo enquanto falo	1	2	3	4	5
23. Tento falar melhor, mas não consigo	1	2	3	4	5
24. Reclamo com colegas por ter que ser eu a falar	1	2	3	4	5
25. Faço caminhadas para me distrair antes de uma apresentação em público	1	2	3	4	5
26. Digo a mim mesmo que a minha apresentação vai dar errado	1	2	3	4	5
27. Acelero a fala para terminar logo	1	2	3	4	5
28. Escuto música para relaxar antes de falar em público	1	2	3	4	5
29. Demonstro irritação quando alguém discorda do que eu estou falando	1	2	3	4	5
30. Assisto vídeos sobre como falar bem	1	2	3	4	5

Famílias de *coping* e itens ECOFAP

**Table:**

Famílias de *coping*	Itens
**FATOR DESAFIO**	
**Busca de informações**: tentativa ativa para aprender mais sobre a situação. Ler, estudar, perguntar aos demais.	1, 11, 30
**Resolução de problemas**: tentativa ativa de modificar a situação ou suas consequências. Planejar estratégias, ações instrumentais.	3
**Autoconfiança:** tentativa ativa de diminuir a angústia emocional. Regulação comportamental e autofala positiva.	7, 18, 22
**Acomodação:** tentativa ativa de redirecionar a atenção para longe da situação estressora, ocupando-se com atividades prazerosas ou modificando a visão sobre a situação. Restruturação cognitiva, aceitação, minimização.	25, 28
**Busca de apoio:** tentativa ativa de usar recursos sociais disponíveis para lidar com a situação estressora por meio de busca de contato, ajuda, referenciamento social.	5, 15, 20
**Negociação:** tentativa ativa de encontrar novas opções, elaborar uma solução de compromisso entre as prioridades individuais e as restrições da situação, estabelecer novos objetivos. Troca.	10
**FATOR AMEAÇA**	
**Desamparo:** o indivíduo se considera impotente frente à situação. Passividade, dúvida, desânimo	12, 23, 19
**Delegação:** o indivíduo tem pena de si ao avaliar que não tem recursos suficientes para lidar com o evento estressor. Reclamação, autoculpa, lamentação.	6, 8, 24
**Fuga:** tentativa ativa para libertar-se da situação e das dificuldades relacionadas a ela. Pessimismo, afastamento mental.	2, 4, 27
**Isolamento:** tentativa ativa de evitar as pessoas ou impede que elas saibam sobre como se sente frente a situação. Afastamento social, evitação dos demais, solidão.	13,14, 17
**Submissão:** tentativa ativa de manter o foco passivo e repetitivo nos aspectos negativos e prejudiciais da situação. Rigidez, ruminação, pensamentos intrusivos.	16, 21, 26
**Oposição:** tentativa ativa de usar comportamentos de raiva, explosão, para remover obstáculos impostos pela situação estressora. Agressão, explosão, culpar os outros.	9, 29

**Correção da ECOFAP:** Calcula-se o somatório simples de cada fator:

**Desafio:** itens 1, 3, 5, 7, 10, 11, 15, 18, 20, 22, 25, 28 e 30 (13 itens, pontuação máxima 65)

**Ameaça:** itens 2, 4, 6, 8, 9, 12, 13, 14, 16, 17, 19, 21, 23, 24, 26, 27 e 29 (17 itens, pontuação máxima 85)
